# Target-Specific Exosome Isolation through Aptamer-Based Microfluidics

**DOI:** 10.3390/bios12040257

**Published:** 2022-04-18

**Authors:** Zixuan Zhou, Yan Chen, Xiang Qian

**Affiliations:** 1 Tsinghua-Shenzhen International Graduate School, Tsinghua University, Shenzhen 518055, China; zhouzx19@mails.tsinghua.edu.cn; 2 Shenzhen Institutes of Advanced Technology, Chinese Academy of Sciences, Shenzhen 518055, China; yan.chen@siat.ac.cn

**Keywords:** aptamer, exosome, isolation, CD63, PTK7, microfluidics

## Abstract

Exosomes (30–100 nm in diameter) are a group of cell-derived membrane vesicles, packaged as valuable cargo with lipid, proteins, and genetic materials from their parent cells. With the increasing interest in exosomes for diagnostic and therapeutic applications, the rapid isolation of pure exosome populations has become a hot topic. In this paper, we propose modified microchannels with aptamer in a microfluidics system for rapid and efficient isolation of exosomes by targeting exosome-carrying CD63 and PTK 7. The capture efficiency in surface-modified channels reaches around 10^7^–10^8^ particles/mL in 20 min, and purified exosomes with reliable size can be achieved.

## 1. Introduction

Different cell types are capable of secreting extracellular vesicles in vitro [1]. Extracellular vesicles (EVs) (30–1000 nm in diameter) are mixed populations of cell-derived membrane structures. They more than just carry wastes, but play an important role in transcellular communication [2,3]. According to biogenesis, or in other words, intracellular origin, EVs have been sorted into two classes, exosomes and other EVs [4]. Exosomes generally refer to 30–100 nm EVs formed by the inward budding of the endosomal membrane during maturation of multi-vesicular endosomes. Endosomes are generated by inward budding from the plasma membrane, and they release exosomes through the fusion with plasma membrane [5]. An exosome contains a specific composition of proteins, mRNA, miRNA, and DNA that carries coding information to communicate between the exosome-producing cell and the target cell, making them attractive candidates for circulating disease biomarkers for clinical application [6,7]. Despite these promising attributes, a major source of ongoing confusion is how to build solid methods for isolating exosomes from mixed EV populations, since all EVs share a similar appearance, overlapping range of size, and often common protein composition, making it difficult to make a clear distinction [8]. Fortunately, however, exosomes bear specific surface molecules such as tetraspanins CD63 and protein tyrosine kinase 7 (PTK7), which can be considered as genuine markers of interest [9]. CD63 is a kind of cell surface-associated membrane protein which is abundantly presented on the exosome surface [10]. PTK7 is a transmembrane receptor that has been reported as present on exosomes and plays an important role in cell-to-cell communication [11]. 

Reported exosome isolation techniques mainly include gold standard–differential ultracentrifugation [12], sequential centrifugal ultrafiltration [13], immuno-beads, polymeric precipitation [14], and size-exclusion chromatography (SEC) [15], requiring dedicated laboratory instruments and time-consuming operations which challenge the experimental convenience [16]. Successful isolation requires reliable characterization to prove it. Transmission electron microscopy (TEM) and scanning electron microscopy (SEM) have been well documented to observe the morphology of exosome. Nanoparticle tracking analysis (NTA) offers the additional ability to measure size distribution and concentration of exosomes [17].

Microfluidics, with the advantage of being a portable, cheap alternative, is employed as a platform in the isolation of exosomes. Generally, the strategies applied for the isolation of exosomes in a microfluidics system include the immunoaffinity-based method, dynamic-based method, and size-based methods [18]. In 2021, Yu et al. developed the ExoSD microfluidic chip endowed with immunomagnetic capture beads to achieve exosomes isolation [19]. In 2014, Kanwar et al. fabricated an ExoChip functionalized with antibodies to isolate exosomes from serum samples [20]. In 2018, Smith et al. created nanoscale deterministic lateral displacement (DLD) arrays on microfluidic channel to achieve collection of EVs without the further step of isolating exosomes [21]. In 2013, filtration-based microfluidics fabricated by Wand et al. achieved the trapping of EVs on nanowire-on-micropillars [22].

Aptamer, a single-stranded DNA or RNA oligonucleotide with complex three-dimensional shape, often contains 15–60 nucleotides. It is generated from a large random sequence pool via in vitro selection based on its specific recognition for targeting molecules; moreover, it can be tailored for a specific target. Chemical modification can enhance activity or stability of aptamers [23,24]. Aptamers, also called chemical antibodies, are usually compared with antibodies, since both molecules function as affinity reagents [25]. The binding domain between the surface protein and oligonucleotide aptamers is electrostatic attraction, hydrogen bond, and van der Waals force. Since the phosphate backbone of DNA is negatively charged, it can contribute more oxygen atoms, in other words, more electrons, when binding to positively charged amino acids, making specific adsorption more stable. Aptamers provide great promise for isolating and detecting EVs in affinity applications [26].

There is not much literature concerning microchannels modified with aptamer in microfluidic chips to achieve the purpose of separating exosomes. Aptamer related exosome applications focus mainly on exosome detection [27,28,29]. Recently, studies have usually applied aptamers to target EVs based on magnetic beads. These works are developed from mature immunomagnetic magnetic beads’ (IMB) separation. Aptamers act as the role of antibodies in IMB. In 2019, Zhang et al. developed DNA aptamer-based magnetic isolation process for EV’s isolation. Cell culture medium or plasma is mixed with biotin labelled CD63 aptamer and then separated using streptavidin modified magnetic beads. NTA characterization of isolated EVs showed 1.4 × 10^7^ particles/mL capture efficiency in 90 min [30]. However, in aptamer-IMB there exists the problem of not eluting independent EVs from beads. In 2020, Song et al. developed a magnetic bead-based exosome immunoaffinity separation system by using CD63 targeting aptamer. NTA results showed that this method captures 8.37 × 10^7^ exosomes particles from 10 mL MDA-MB-231 cell culture mediate in 60 min. The native state exosomes were eluted using NaCl elution [31].

In this paper, we present aptamer-affinity microfluidics to endow the isolation platform with targeting capability. The chip, which is made from polydimethylsiloxane (PDMS) and glass, implements exosomes isolation based on immobilized DNA-aptamers in microchannels. Two exosomes-carried biomarkers, CD63 and PTK7, which are ubiquitously presented on most exosomes, serve as targets for specific capture by Apt-CD63 [32] and Apt-PTK7 [9], respectively. These aptamers are immobilized via the biotin–avidin–desthiobiotin link on a glass slab. The chip benefits from a combination of filtration-based apparatus and aptamer-affinity based apparatus. The microfilter in the inlet achieves the ability of starting from cell culture supernatant without previous treatment, since it in attempts to filtrate cell debris, bacteria, or PDMS residues before aptamer-affinity reaction. The cell culture supernatant sample is composed of large-sized impurities, such as cell debris and proteins, and small-sized particles, such as exosomes and other EVs. The aim of the aptamer-based apparatus is to capture exosomes from mixed EVs populations. According to reviews, exosomes contain CD63 and PTK7 distinct from other EVs, such that we chose these two surface biomarkers as aptamer targeting proteins and also chose them as ELISA proteins in the following exosome characterization experiment.

## 2. Materials and Methods

### 2.1. Cancer Cells Culture 

The lung cancer cell lines A549 were obtained from the Shanghai cell bank of the Chinese Academy of Science. The cells were cultured at 37 °C in a humidified atmosphere of 5% CO_2_ in cell incubator (Thermo Fisher Scientific, Waltham, MA, USA). The cell culture medium contains 90% RPMI Medium 1640 supplemented with 10% fetal bovine serum (FBS) depleted of EVs and 1% penicillin-streptomycin (PS). The freezing medium contains 90% FBS and 10% dimethylsulfoxide (DMSO). Phosphate buffered saline (PBS), FBS, PS, and 0.25%Trypsin-EDTA, RPMI Medium 1640 were purchased from Thermo Fisher Scientific, Waltham, MA, USA. For repeated experiments using the same batch of the cell lines, the slow-freezing and rapid-thawing method was utilized. When the cells reach 80% to 90% coverage, passaging should be operated. Passage 6–8 cell culture supernatant was prepared for experiments.

### 2.2. Working Principle and Fabrication Processes of the Aptamer-Based Microfluidics

**Figure 1:**
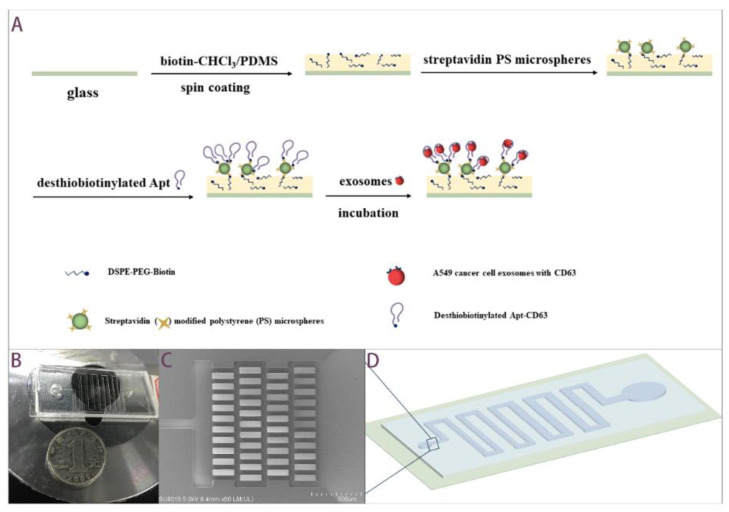
Aptamer-based exosomes isolation microfluidics. (**A**) Immobilization of aptamer onto glass surface for EVs capture. (**B**) Prototype of PDMS/glass chip in comparison with a 1 yuan RMB coin. (**C**) SEM image of micropillars in inlet. The scale bar is 500 μm. (**D**) Schematic diagram of PDMS/glass microfluidics.

Our aptamer-based microfluidic chip applies the principal of using the biotin–avidin–desthiobiotin binding link to immobilize aptamer on the surface of the channel. DSPE–PEG–Biotin are embedded in the PDMS prepolymer on glass slab by mixing with chloroform, as reported by Huang et al. [33]. Aptamers modified with desthiobiotin, an analogue of biotin, are immobilized by the lower binding affinity with avidin. Desthiobiotin instead of biotin binds with avidin to facilitate easier release of aptamer-conjugated exosomes [34]. Figure 1A displays fabrication steps of functionalized glass slab. A filtration-based apparatus is conducted in order to hinder cell debris and PDMS residues to enter into the downstream. The affinity-based apparatus possesses the ability for discrimination between the exosomes of interest and other EVs in the fluids. Figure 1B and Figure 1D show a photo of PDMS/glass microfluidics and a schematic diagram, respectively. In order to reduce the flow resistance, the microchannel is gradually widened from 100 μm to 1000 μm at the end. Figure 1C shows a SEM image of microfilter in inlet, correspondingly labeled in Figure 1D. The gap between micropillars is around 50 μm.

The chip was composed of PDMS top slab and glass bottom slab. The PDMS slab was fabricated by soft lithography techniques. The layout of the channel structure was designed in AutoCAD (Autodesk Inc., San Rafael, CA, USA) and printed it on a film (Shenzhen Qingyi Photomask, Shenzhen, China). The pattern on the film was transferred to a silicon wafer with photoresist via lithography using UV-365 alignment (URE-2000/35, Institute of Optics and Electronics, Chinese Academy of Science, Chengdu, China). We adopted an ultrathick photoresist, SU8-2050 (Nippon Kayaku, Tokyo, Japan), and the depth was 100 μm. The silicon wafer with patterned photoresist was utilized as a master mold in molding process. PDMS prepolymer and curing agent mixture (10:1 volume ratio) (Sylgard 184 reagent, Dow Corning, Midland, MI, USA) was poured into master mold and cured in an oven at 80 °C for 1 h. The elastic PDMS slab was obtained by peeling. The bottom slab was a functionalized cover glass. A 1 mL 5 mg/mL biotin-chloroform solution was added to 3 g PDMS prepolymer and the curing agent mixture (10:1 volume ratio). The mixture covered, as thinly as possible, over the entire glass via spin coating step. Otherwise, a thicker layer would cause blocking during the subsequent bonding step. With the limitation of experimental conditions, the maximum speed we used was 1800 rpm. The top slab of the flow channel was then put on the mixture-coated cover glass before the latter was cured at 37 °C overnight. 

DSPE–PEG–Biotin and Streptavidin modified green fluorescent polystyrene microspheres (5 μm) were purchased from Xi’an ruixi Biological Technology, Shanghai, China. Apt-CD63: 3′ Desthiobiotin-CAC CCC ACC TCG CTC CCG TGA CAC TAA TGC TA-5′ [32] and Apt-PTK7: 3′ Desthiobiotin-TTT TTT TAT CTA ACT GCT GCG CCG CCG GGA AAA TAC TGT ACG GTT AGA-5′ [9] were synthesized by Sangon Biotech, Shanghai, China with reversed-phase HPLC purification. Streptavidin and biotinylated aptamer were introduced into device one after another by connecting flow control kit (MFCS-EZ, Fluigent, Okabé, France) from the inlet. After the channel surface was modified with aptamers, cell culture supernatant was introduced into the device for 10 min incubation for exosomes isolation, and captured exosomes were expected to be eluted into PBS washing buffer.

### 2.3. Exosomes Isolation

**Figure 2:**
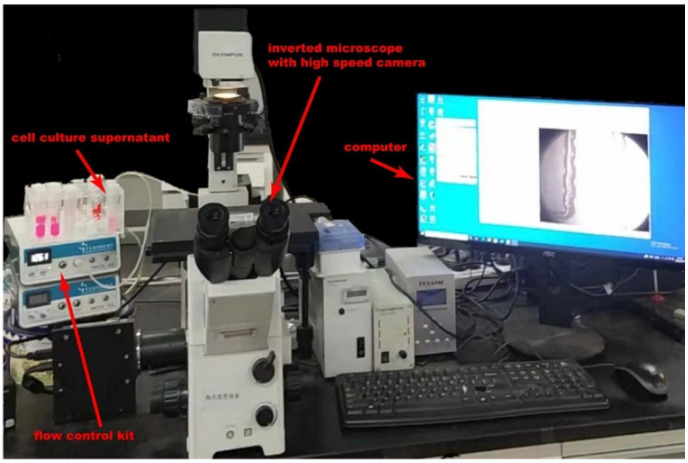
The experiment setup.

First, 4 mL A549 Cell culture supernatant was run and incubated through our device for exosomes capture for approximately 10 min and discharged with a pressure pump. Trapped lipid vesicles were recovered intact in 4 mL PBS buffer for 10 min. The whole experiment setup is shown in Figure 2. By pumping nitrogen gas into the flow control kit, a pressure-diven flow was created in microchannel. The flow velocity could be controlled directly by manipulated gas pressure on software. For comparison, another 4 mL A549 Cell culture supernatant was purified by using exoEasy Maxi Kit (Qiagen, Hilden, Germany) and exosomes were eluted into 4 mL buffer.

### 2.4. Exosomes Characterization

#### 2.4.1. Morphological Characterization of Chip-Isolated Exosomes

For TEM analysis, 10μL exosomes/PBS solution were fixed with 10 μL 2% Paraformaldehyde (PFA) (Dalian Meilun Biological Technology, Dalian, China) on a formvar–carbon coated EM grid (Beijing XXBR Technology, Beijing, China) for 30 min at room temperature [35]. The grid was floated on a drop of 2% Phosphotungstic acid hydrate (PAH) (Shenzhen Ziker Biological Technology, Shenzhen, China) three times for several seconds and used a filter paper strip to absorb excess liquid. After washing twice with PBS, the fixed exosomes were dehydrated with a descending sequence of ethanol (40%, 60%, 80%, 96–98%) [36]. Exosomes were observed under low beam energies as 1.5 kV at 1 mA by using TEM (Tecnai G2 F30). 

For AFM analysis, 10 μL exosomes/PBS were fixed with 10 μL 2% PFA on a freshly cleaved mica sheet for 30 min. After washing with PBS, a filter paper strip was used to absorb excess liquid from the edge of the mica sheet [37]. The sample was tested under Peak Force Modulation at a scan rate of 0.9 Hz, and the vibration amplitude of the oscillating cantilever was 300 kHz by using AFM (Bruker Dimension Icon). 

#### 2.4.2. Size Distribution and Concentration Characterization of Isolated Exosomes

The concentration and size distribution of particles in exosomes collected solutions were analyzed by using NanoSight NS300 (Malvern Instruments, Malvern, UK) equipped with video capture and particle-tracking software via measuring the rate of Brownian motion of particles. 

#### 2.4.3. Exosomes-Carried Proteins CD63 and PTK7 ELISA Characterization of Isolated Exosomes 

100 μL conjugate reagent was added to each exosome sample and then incubated at 37 °C for 1 h. The exosome sample contains 10 μL exosomes recovery and 40 μL sample dilution, which means that the sample’s final dilution factor is five-fold. After clearing, the samples were incubated with chromogen solution at 37 °C for 15 min and, subsequently, absorbance was read spectrophotometrically at a wavelength of 450 nm. Human Cluster of differentiation (CD63; TSPAN30) ELISA Kit and Human Protein Tyrosine Kinase 7(PTK7) ELISA Kit were purchased from China Jiangsu Meibiao Biotechnology, Yancheng, China.

## 3. Results and Discussion 

### 3.1. Morphological Characterization of Chip-Isolated Exosomes 

**Figure 3:**
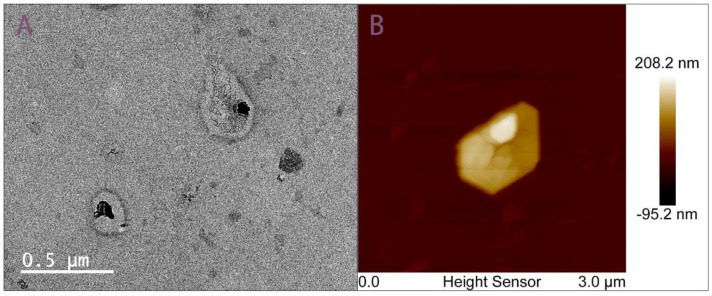
TEM and AFM imaging of exosomes isolated by aptamer-based microfluidics. (**A**) TEM image of EVs isolated by microfluidics presents a distorted cup-shaped morphology and uniform unimodal size distribution following 200 nm filtration. (**B**) AFM image EVs isolated by microfluidics presents a distorted cup-shaped morphology.

Intuitive characterization of exosomes relies on microscopy techniques with high resolution. Chip-isolated exosomes are observed under TEM and AFM after specialized staining protocols. In Figure 3, microscopic morphological diagnosis demonstrates exosomes captured by chip show intact cupped-croissant morphology in 100–200 nm that are consistent with those previously reported [36,37]. We did not use biological sample-specific probes, so the edges of exosomes are not smooth, which is due to the limited experimental conditions (Figure 3B). Although this AFM image is not particularly ideal, it is still meaningful because it provides further evidence that the exosomes are cup-shaped. Since the AFM image of exosomes captured by using exoEasy Maxi Kit has not been successfully obtained, it failed to be compared with the morphology of exosomes isolated from two methods here.

### 3.2. Concentration and Size Distribution Characterization of Isolated Exosomes 

**Figure 4:**
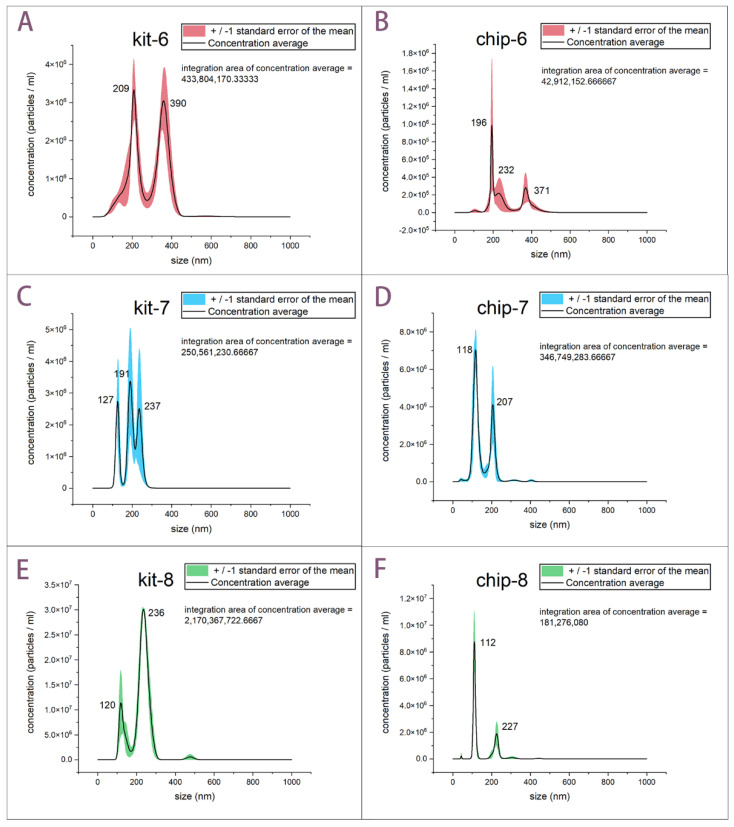
NTA of exosomes by using aptamer-based microfluidics and exoEasy Maxi Kit. (**A**) Averaged concentration/size distribution of exosomes collected from cell culture supernatant (passage-6) by using exoEasy Maxi Kit. (**B**) Averaged concentration/size distribution of exosomes collected from cell culture supernatant (passage-6) by using our device. Error bars indicated +/− standard error of the mean. (**C**,**D**) are the data graphs from cell culture supernatant (passage-7). (**E**,**F**) are the data graphs from cell culture supernatant (passage-8).

TEM and AFM can illustrate the morphological appearance of single exosome particle clearly. However, according to the limited field of view, we cannot acquire the size distribution of exosome populations by enumerating all the TEM pictures. Thus, to demonstrate the ability and efficiency of our aptamer-based microfluidics for isolation of exosomes, we used cell culture media of different passages as a single source of a specific exosome population. Passage 6–8 cell culture supernatant was prepared for NTA characterization. Isolated exosomes using exoEasy Maxi Kit served as a reference for verifying the performance of our device. The NTA results are illustrated in Figure 4. There are six samples in Figure 4, and the number “6” in Figure 4A,B represents NTA results of kit and our chip by using passage 6 cell culture supernatant, respectively. By the analogy, “kit-7” and “chip-7” means NTA result of kit and our chip by using passage 7 cell culture supernatant, respectively, and the same is true for “kit-8” and “chip-8” in Figure 4. Each sample was run three times and the standard error is calculated by the following formula: Standard error = STDEV.S/ SQRT (3). The particle population from NTA shows that enrichment of exosomes by using the kit and our chip is almost identical. It should be noted that chips’ peaks show a smaller mean diameter compared to those of the kit under the circumstance of the same passage of cell culture supernatant. This indicates that exosomes isolated by using aptamer-based microfluidics are closer to the theoretical size range of exosomes, since many reviews have reported that exosomes bear smaller diameters (30–100 nm) than other EVs (100–1000 nm), which was pointed out in the introduction section. Exosomes isolated by using our chip show smaller size than those from the exoEasy Maxi Kit, so our aptamer-affinity microfluidics indeed can isolate reliable exosomes compared with the exoEasy Maxi Kit, while kit-isolated exosomes are mixed with other larger EVs. 

Combining the results of morphology and size distribution, we confirmed that our aptamer-based chip can conduct a better approach to isolate exosomes with ideal size distribution and perfect shape. Integrated results show the capture efficiency of around 10^8^–10^9^ particles/mL in 20 min by using exoEasy Maxi Kit. Our procedure is satisfactory with regard to capture efficiency of 10^7^–10^8^ particles/mL in 20 min (Figure 4). We estimated the times of each experiment were both around 20 min.

### 3.3. Exosomes-Carried Proteins CD63 and PTK7 ELISA Characterization of Isolated Exosomes

Additionally, to fully demonstrate the target-specific capturing of our aptamer-based microfluidic chip, quantitative enzyme-linked immunosorbent assay (ELISA) for exosome-carried marker CD63 and PTK7 was adopted. Considering that exosomes contain CD63 and PTK7 distinct from other EVs, they were chosen to quantitatively confirm how many particles recovered were true exosomes. We have mentioned above that these two proteins are also our aptamer targeting proteins. According to the ELISA kit use instruction, the standard density is used as the horizontal and the O.D value for the vertical. Linear regression analysis shows the positive correlation between O.D value and standard CD63 concentration, with intercept value ranging in 0.173 ± 0.078 and slope value ranging in 0.375 ± 0.027 (Figure S1A). There is a positive correlation between O.D value and standard PTK7 concentration, with intercept value ranging in 0.103 ± 0.022 and slope value ranging in 0.218 ± 0.005 (Figure S1B). Thus, the concentration of CD63 and PTK7 in the previously mentioned six samples can be determined by comparing the O.D value of the samples to the standard curve, respectively (Table S1).

In Table 1, we found that each exosome’s recovery by using our aptamer-based microfluidics has a higher concentration of CD63 and PTK7 compared with its counterpart (kit-sample). The concentration difference is greater than 1 pg/mL. The comparison results are reliable due to the limit of detection being less than 0.1 pg/mL. The higher protein concentration, the more determinate exosomes in the chip recovery sample, because these two proteins are carried on the exosome’s surface. The exoEasy Maxi kit claimed to adopt chromatographic spin columns for purifying exosomes and other EVs, which seems unreliable for a purification of exosomes [38]. Although the kit has a superior EVs yield (10^8^–10^9^ particles/mL) to our chip (10^7^–10^8^ particles/mL), our device endows properties of targeting and specificity that will allow one to enrich more exosomes from a complex EVs population. Further work is necessary to understand the mechanism by which our device endows properties of targeting and specificity to enrich more exosomes from complex EVs population. Table 1 summaries particle populations of “kit-6” and “kit-8” is an order of magnitude higher than those data coming from “chip-6”and “chip-8”, respectively. Nevertheless, chip samples contain more exosomes of interest even when counted by relatively moderate particles. These imply that aptamer has a great potential in affinity application, such as capturing pollutant organic macromolecules, and not only in its use for targeting specific exosomes. Unfortunately, it should be pointed out that we cannot determine that only exosomes are present in chip recovery, because other EVs may also contain CD63 or PTK7, since the endosomes (the origin of exosomes) and EVs are both budding from the plasma membrane. CD 63 and PTK7 are highly enriched on the plasma membrane.

In summary, via aptamer-based microfluidics combined with microfilter apparatuses in our chip, we isolated intact exosomes from A549 cell culture supernatant, displaying not only high capture efficiency but also convenient operation. Our chip is satisfactory, with a capture efficiency of 10^7^–10^8^ particles/mL in 20 min compared with that of 10^8^–10^9^ particles/mL in 20 min by using a commercial kit. The cell culture supernatant rapidly contacts with aptamer modified in microchannels as it flows through, which greatly reduces the time by replacing the method of mixing cell culture supernatant with aptamer–IMB. The time is shortened without affecting the performance of aptamer, because this exosome’s isolation is based on the microfluidics. Furthermore, before supernatants enter into the aptamer-based apparatus, the microfilter apparatus in our chip can filtrate impurities such as cell debris. However, when we use the exoEasy Maxi Kit, supernatants require extra operations to filter particles larger than 0.8 μm by using syringe filters. Importantly, the aptamer-based apparatus has the ability to capture exosomes from mixed EVs populations. In theory, exosomes show smaller diameters (30–100 nm) than other EVs (100–1000 nm). According to NTA, exosomes isolated by using our chip are closer to the theoretical size of exosomes than those from the exoEasy Maxi Kit. Therefore, our aptamer-affinity microfluidics can indeed isolate reliable exosomes compared with the exoEasy Maxi Kit, while there are other larger EVs mixed in with the kit recoveries. The results of ELISA showed that CD63 and PTK7 carried by exosomes are more abundant in chip recoveries, which further confirmed that the exosomes isolated by our aptamer-based microfluidics are more authentic.

## 4. Conclusions

Purifying the exosome populations from other EVs or other interferences from biological samples is still an open problem. In this paper, we proposed an aptamer-based microfluidics for exosome isolation, which possesses the advantages of being easy-to-use, high efficiency, and target specific. We have demonstrated the capture ability of exosomes in such aptamer-affinity based microfluidic chips from cell culture supernatants as high as 10^7^–10^8^ particles/mL with reliable size. In this way, our chip combining an aptamer-based apparatus with a filtration-based apparatus achieves the ability of starting from cell culture supernatant without pre-treatment, potentiating a convenient experiment. The specific aptamer targeting exosome-carried proteins CD63 and PTK7 achieved reliable exosomes isolation. It should be pointed out that we cannot qualify how many aptamers are modified on the microchannel, which is something we need to improve. We simply employed aptamers combined with the microfluidics platform, demonstrating that the aptamer has great potential to specifically capture exosomes. Furthermore, such microfluidics immobilized with aptamers can be customized to different targeted sets of the user’s requirements, which will contribute to the development of enriching particles for clinical diagnosis or drug delivery in the future.

## Figures and Tables

**Table 1:** Protein concentration of EVs recovery.

	Sample	Kit-6	Chip-6	Kit-7	Chip-7	Kit-8	Chip-8
CD63	O.D value (dilution 5-fold)	0.5106	0.581	0.5107	0.5316	0.5025	0.5839
	CD63 concentration (pg/mL)	80.39	93.48	80.41	84.29	78.88	94.01
PTK7	O.D value (dilution 5-fold)	0.541	0.548	0.518	0.561	0.568	0.587
	PTK7 concentration (pg/mL)	10.032	10.19	9.51	10.49	10.65	11.09
Particles/mL		4.3 × 10^8^	4.3 × 10^7^	2.5 × 10^8^	3.4 × 10^8^	2.1 × 10^9^	1.8 × 10^8^

## Data Availability

Not applicable.

## Supplementary Materials

The following supporting information can be downloaded at: https://www.mdpi.com/article/10.3390/bios12040257/s1, Figure S1: Standard curve of ELISA, Table S1: Protein concentration of EVs recovery.
