# Supplementary material for: Target-Specific Exosome Isolation through Aptamer-Based Microfluidics
Source: Biosensors (Basel). 2022 Apr 18;12(4):257. doi: 10.3390/bios12040257 (PMC9027373; doi:10.3390/bios12040257)
Supplement: Supplementary file 1 [file biosensors-12-00257-s001.zip › biosensors-1540743-supplementary.pdf]

biosensors

Supplementary Materials

Target-Specific Exosome Isolation through Aptamer-Based Microfluidics

Zixuan Zhou [1], Yan Chen [2] and Xiang Qian [1,*]

[1] Tsinghua-Shenzhen International Graduate School, Tsinghua University, Shenzhen 518055, China; zhouzx19@mails.tsinghua.edu.cn
[2] Shenzhen Institutes of Advanced Technology, Chinese Academy of Sciences, Shenzhen 518055, China; yan.chen@siat.ac.cn
* Correspondence: qian.xiang@sz.tsinghua.edu.cn

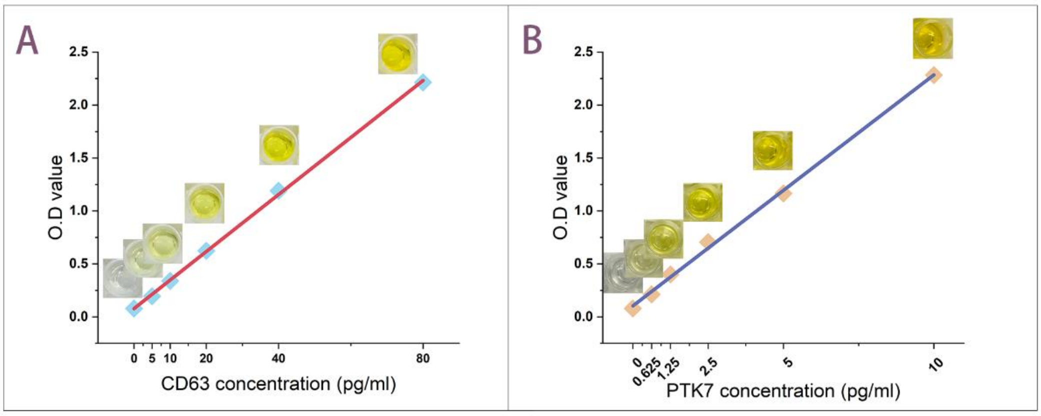

Figure S1. Standard curve of ELISA. (A) The correlation between O.D value and CD63 concentration. (B) The correlation between O.D value and PTK7 concentration

Table S1. Protein concentration of EVs recovery.

	Sample	Kit-6	Chip-6	Kit-7	Chip-7	Kit-8	Chip-8
CD63	O.D value (dilution 5-fold)	0.5106	0.581	0.5107	0.5316	0.5025	0.5839
	Color (dilution 5-fold)	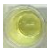	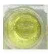	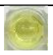	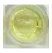	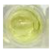	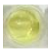
	CD63 concentration (pg/ml)	80.39	93.48	80.41	84.29	78.88	94.01
PTK7	O.D value (dilution 5-fold)	0.541	0.548	0.518	0.561	0.568	0.587
	Color (dilution 5-fold)	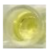	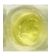	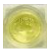	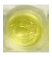	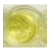	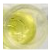
	PTK7 concentration (pg/ml)	10.032	10.19	9.51	10.49	10.65	11.09
integrated area		433,804,170	42,912,153	250,561,231	346,749,284	2,170,367,723	181,276,080
particles/ml		4.3×10^8	4.3×10^7	2.5×10^8	3.4×10^8	2.1×10^9	1.8×10^8
